# Composite Chronic Lymphocytic Leukemia/Small Lymphocytic Lymphoma and T-Prolymphocytic Leukemia Presenting with Lymphocytosis, Skin Lesions, and Generalized Lymphadenopathy

**DOI:** 10.1155/2019/4915086

**Published:** 2019-03-03

**Authors:** Ali Sakhdari, Guilin Tang, Lawrence E. Ginsberg, Cheryl F. Hirsch-Ginsberg, Carlos E. Bueso-Ramos, L. Jeffrey Medeiros, Roberto N. Miranda

**Affiliations:** ^1^Department of Hematopathology, The University of Texas MD Anderson Cancer Center, Houston, TX, USA; ^2^Department of Diagnostic Radiology, The University of Texas MD Anderson Cancer Center, Houston, TX, USA; ^3^Department of Laboratory Medicine, The University of Texas MD Anderson Cancer Center, Houston, TX, USA

## Abstract

Chronic lymphocytic leukemia (CLL) is the most common type of leukemia in Western countries with an incidence of 3-5 cases per 100,000 persons. Most patients follow an indolent clinical course with eventual progression and need for therapy. In contrast, T-prolymphocytic leukemia (T-PLL) is a rare type of T-cell leukemia with most patients having an aggressive clinical course and a dismal prognosis. Therapies are limited for T-PLL patients and there is a high relapse rate. Morphologically, the cells of CLL and T-PLL can show overlapping features. Here, we report the case of a 61-year-old man who presented with a clinically indolent CLL and T-PLL, initially diagnosed solely and followed as CLL, despite the presence of an associated but unrecognized aberrant T-cell population in blood. After 2 years, the T-PLL component became more apparent with cutaneous and hematologic manifestations and the diagnosis was confirmed by immunophenotypic and cytogenetic analysis. Fluorescence* in situ* hybridization demonstrated an* ATM* deletion in both CLL and T-PLL components. Retrospective testing demonstrated that composite CLL and T-PLL were both present in skin and lymph nodes as well as in blood and bone marrow since initial presentation. This case is also unique because it highlights that a subset of T-PLL patients can present with clinically indolent disease. The concomitant detection of* ATM* mutation in CLL and T-PLL components raises the possibility of a common pathogenic mechanism.

## 1. Introduction

T-prolymphocytic leukemia (T-PLL) is a rare mature T-cell neoplasm that frequently presents with lymphocytosis, hepatosplenomegaly, lymphadenopathy, skin lesions, and serous effusions [1, 2]. The disease is most common in the elderly with a slight predilection for males [3]. Although most cases of T-PLL are clinically aggressive with frequent relapses, resistance to conventional chemotherapeutic modalities, and poor overall survival, a subset of patients with T-PLL initially present with a clinically indolent course [4, 5]. Cases of T-PLL show morphologic and immunophenotypic heterogeneity and therefore integration of clinicopathologic, laboratory, immunophenotypic, cytogenetics, and recently identified molecular features may be needed for proper discrimination from similar T-cell neoplasms that can present in leukemic phase [6, 7]. The introduction of anti-CD52 (alemtuzumab) in the frontline treatment of patients with T-PLL has dramatically increased the rate of complete remission (CR) and overall survival (OS) in this population, although most T-PLL patients ultimately relapse. Allogeneic or autologous stem cell transplantation may have a curative effect [8].

Chronic lymphocytic leukemia/small lymphocytic lymphoma (CLL/SLL) is the most common chronic B-cell leukemia in Western countries with an incidence increasing with age [1, 9, 10]. Most patients with CLL/SLL follow an indolent clinical course and as many as two-thirds of patients do not need treatment at presentation. Untreated patients have a progressive accumulation of leukemic cells in the bone marrow and other lymphoid and nonlymphoid organs [11]. Eventually, symptomatic patients with high-stage disease need therapy at the time of diagnosis or soon after [12, 13]. In addition, immune phenomena are commonly associated with CLL/SLL, including autoimmune manifestations, immunodeficiency, opportunistic infections, and secondary neoplastic disorders [14–16]. Transformation to a more aggressive disease such as large B-cell lymphoma, or less frequently to other types of hematolymphoid malignancies, occurs in a small subset of patients [17–24]. More rarely, and after therapy, patients with CLL may develop a clonally unrelated T-cell lymphoma [22, 25, 26] or alternatively a histiocytic lineage neoplasm in a process called transdifferentiation, on which clonal relatedness can sometimes be demonstrated [27–29]. Herein we report the case of a 61-year-old patient who presented with composite CLL/SLL and T-PLL that was not recognized until the disease was advanced, and in retrospective analysis both disease components were present in different organ systems. Although similar cases have been rarely reported, herein we demonstrate with immunophenotypic markers and FISH probes in tissue sections that both disease components were together since initial presentation and propose a pathogenic mechanism based on the shared mutation of* ATM* gene mutation [30–32].

## 2. Case Presentation

**Figure 1:**
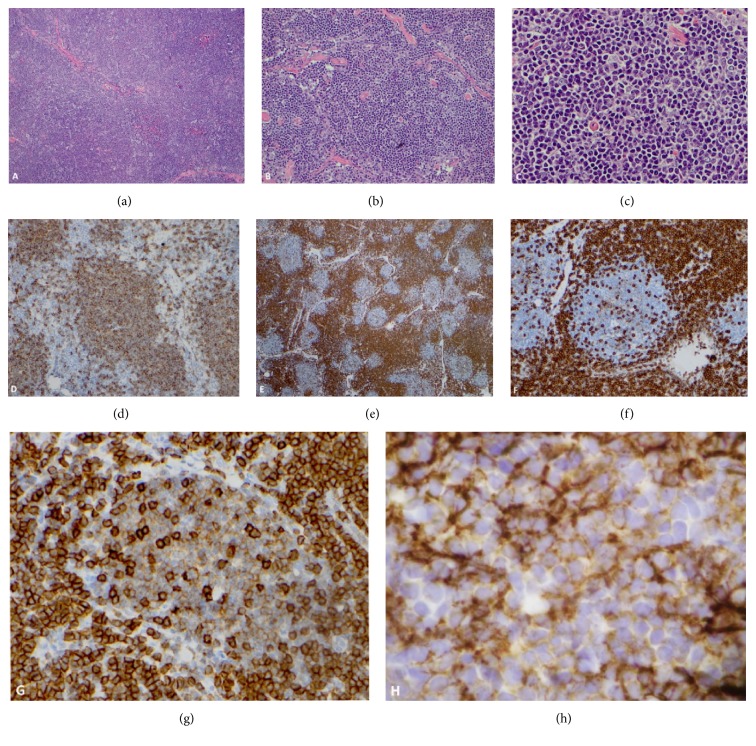
Composite chronic lymphocytic leukemia/small lymphocytic lymphoma (CLL/SLL) and T-prolymphocytic leukemia (T-PLL). This lymph node shows partial effacement of the architecture by a vaguely nodular/follicular proliferation of small-to-medium-sized lymphocytes surrounded by a dense infiltrate of small-to-intermediate-size lymphocytes. Hematoxylin and eosin stain (a–c). Immunohistochemistry shows that the nodular/follicular areas react with the B-cell marker CD20 (d), whereas the interfollicular areas are highlighted with the T-cell marker CD3 (e, f). Note that the follicular areas are also faintly positive with CD5 (g) and dimly positive with CD23 (h), supporting CLL/SLL (h). This distribution of CLL/SLL centered on follicular dendritic cell meshworks highlighted with CD23 is extremely unusual and supports the so-called follicular pattern of CLL/SLL.

A 61-year-old man was diagnosed with prostatic adenocarcinoma on routine work-up for nocturia and back pain in 2015, and a radical prostatectomy with a pelvic lymph node dissection was performed two months later. The lymph nodes were negative for metastatic prostate cancer but, however, showed partial effacement of the nodal architecture. Immunohistochemical studies performed on select lymph nodes showed nodular/follicular areas mainly composed of B-lymphocytes positive for CD20, CD5 (dim), CD23, and BCL2. These lymphocytes were negative for CD3, CD10, and cyclin D1. The interfollicular areas were almost entirely composed of T-lymphocytes expressing CD3, CD5 (bright), CD43, and BCL2. Interestingly, the pattern of CLL/SLL in the lymph node was unusual, as it seemed that the neoplastic cells were restricted to lymphoid follicles, a pattern known as the follicular pattern of CLL/SLL (Figures 1(a)–1(h)). A complete blood count showed a white blood cell (WBC) count of 12.5 × 10^9^/L and flow cytometry immunophenotypic analysis showed that 26% of blood cells had the following immunophenotype: CD20 (+), CD5 (+), CD19 (+), CD22 (+), CD23 (partial +), CD79b (+), CD200 (+) with surface immunoglobulin lambda light chain restriction, supporting a diagnosis of CLL/SLL.

A referral report showed that conventional cytogenetic analysis revealed a complex karyotype and fluorescence* in situ* hybridization (FISH) screen for CLL/SLL revealed del(11q) and del(13q). Mutational status of the immunoglobulin heavy chain (*IGH*) revealed hypermutation of the variable region. The peripheral blood WBC doubling time was estimated at 6 months and hence was determined as not susceptible of chemotherapy. Mutational analysis using next-generation sequencing (NGS) 51-gene panel for hematologic neoplasms showed mutations in* ATM* and* MDM2*.

**Figure 2:**
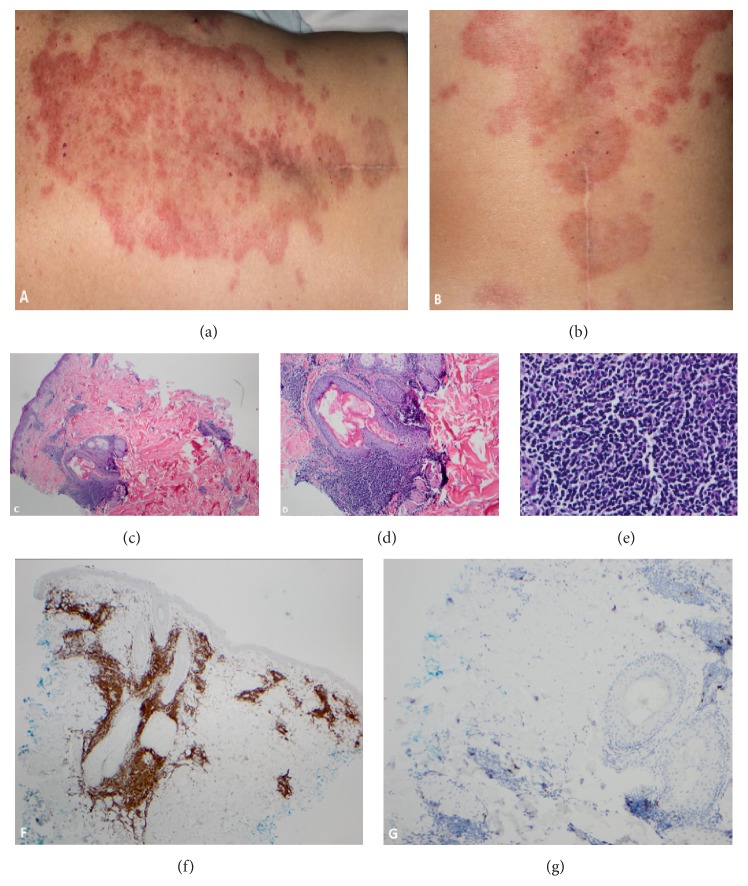
Annular erythematous maculopapular lesions on the upper and lower back (a, b). Dermal infiltration of small-to-medium-sized mature lymphocytes with periadnexal and perivascular distribution (c, d, e). These lymphocytes reacted predominantly with CD3 (f) and CD5 (not shown); only rare lymphocytes were positive for CD20 (g).

Due to the low-stage disease as determined by the lack of any major clinical or laboratory abnormalities, the patient was followed up with observation (“watch and wait”). At the time of initial diagnosis of CLL/SLL in early 2016, the patient noted a skin rash, mainly in the back with a waxing and waning clinical course (Figures 2(a) and 2(b)). Multiple skin biopsies were performed on these lesions diagnosed as superficial and deep dermis small T-cells and rare small lymphocytes with a periadnexal and perivascular distribution; a diagnosis of B-cell lymphoma was excluded (Figures 2(c)–2(g)). In one of these biopsy specimens, polymerase chain reaction- (PCR-) based assay to assess* TRG* revealed a small monoclonal T-cell population in a background of oligoclonal T-cells. PCR for* IGH* in the same specimens was consistently negative (Figures 2(a)–2(g)).

**Figure 3:**
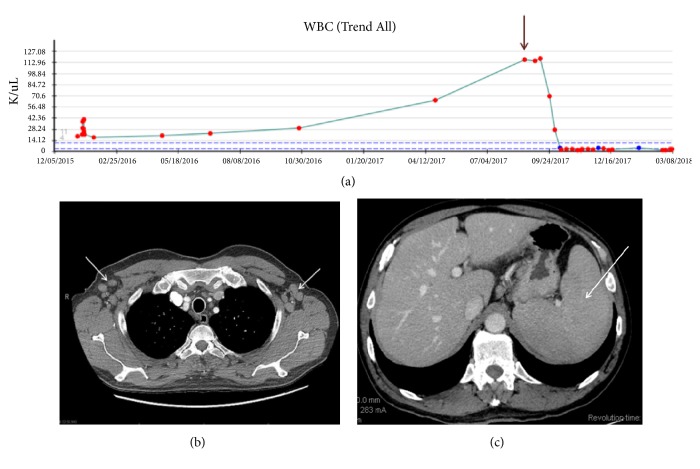
(a) Trending of WBC count from the time of diagnosis in January 2016 to the latest follow-up in March 2018. The arrow points to the WBC count at the time of bone marrow biopsy. It is noted that subsequent therapy led to leukopenia. (b) and (c) Computed tomography (CT) of mid-upper thorax shows multiple enlarged lymph nodes (arrows) in the mediastinum and axilla (b), while CT of the upper abdomen demonstrates a markedly enlarged spleen (arrow) (c).

In August 2017, the patient developed anorexia, fatigue, headache, and drenching night sweats. The rash on his back increased in size while on topical treatment. The peripheral blood WBC count rose to 117 × 10^9^/L, compared with 12.5 × 10^9^/L at diagnosis (Figure 3(a)). A new staging computed tomography (CT) scan showed multicompartmental lymphadenopathy and splenomegaly (Figures 3(b) and 3(c)).

**Figure 4:**
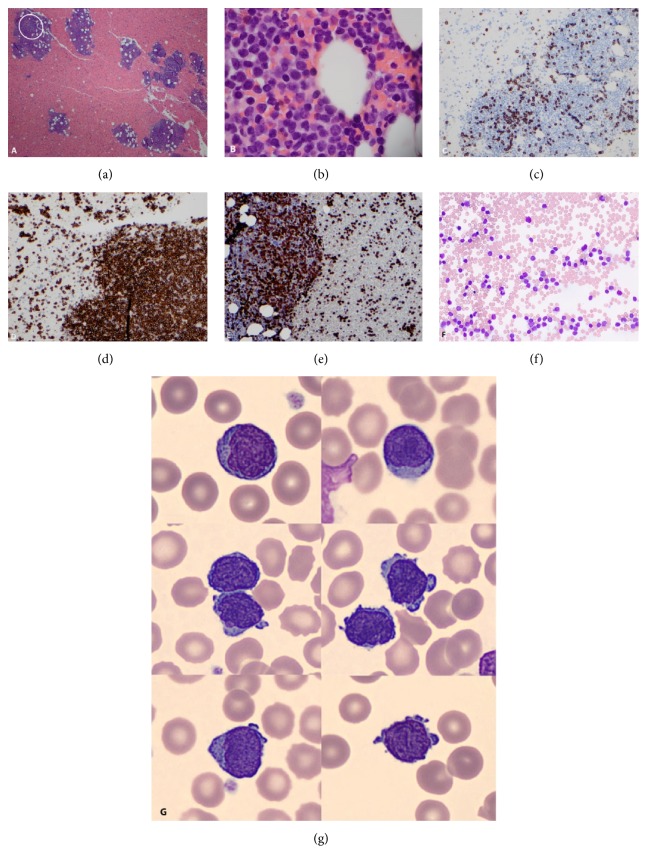
T-PLL in bone marrow biopsy specimen. A bone marrow clot section shows multiple lymphoid aggregates (white circle) composed predominantly of small-to-intermediate-size lymphocytes ((a), low power, (b), high power). The B-cell marker CD20 highlights scattered small lymphocytes (c). The T-cell marker CD3 highlights most lymphocytes in the lymphoid aggregates (d, e). Bone marrow aspirate smear shows numerous small-to-intermediate-size lymphocytes with scant cytoplasm displaying small projections and central nuclei with distinct nucleoli (f). The spectrum of T-PLL lymphocytes in the peripheral blood is shown (g); lymphocytes are predominantly small to intermediate in size with oval to slightly irregular nuclei and distinct nucleoli. The cell contours show blebs, while the cytoplasm is bluish and eccentric. Similar peripheral blood features were noted at initial presentation 3 years before.

The clinical diagnosis of progression of CLL/SLL was established and bone marrow aspiration and biopsy were performed before the initiation of planned therapeutic regimen of B-cell receptor inhibitor ibrutinib and anti-Bcl2 monoclonal antibody venetoclax. The biopsy specimen showed ~80% cellular bone marrow involved by three distinct aberrant cell populations: (a) T-PLL, representing ~70% of bone marrow cells, (b) minor population of CLL, representing 1.2% of bone marrow cells, and (c) CD5-negative small monotypic B-cells, representing 2.8% of bone marrow cells. The concurrent peripheral blood smear showed predominance of small-to-medium-sized lymphocytes with irregular nuclear contours, clumped chromatin, and conspicuous nucleoli (Figures 4(a)–4(g)).

Conventional cytogenetic analysis showed a complex karyotype: 40~45,X,-Y,add(3)(q29),-11,add(12)(p13),-13,inv(14)(q11.2q32),-15,der(15;22)(q10;q10),-16,-18,-19,-20,+6~10mar[cp3]/46,XY[17]. FISH analysis showed* TCL1* rearrangement and deletions of* ATM*, D13S319 locus, and* LAMP1*. Gene clonality assays showed both monoclonal* TRB* and* IGH* rearrangements. Amplicon-based targeted next-generation sequencing (NGS) assay performed using 28-gene panel on genomic DNA extracted from bone marrow aspiration showed* ATM* mutation (NM_000051.3(ATM):c.8078_8080del p.A2693del) at high frequency (variant allele frequency [VAF] of 28%) and* MYD88* (NM_002468.4(MYD88):c.794T>C p.L265P) gene mutation at a very low frequency (VAF of <5%). Clinical and pathologic features of CLL/SLL and T-PLL as seen in our patient are summarized in Table 1.

The patient was diagnosed with composite T-PLL and CLL/SLL. The patient was started on alemtuzumab (total of 3 months of treatment with 30 milligrams 3 times weekly with continuous venous infusion) with excellent clinical and laboratory response showing reduction in size of mediastinal, hilar, axillary, abdominal, pelvic, and inguinal adenopathy. A substantial reduction in previous splenomegaly was observed (Figure 3).

**Figure 5:**
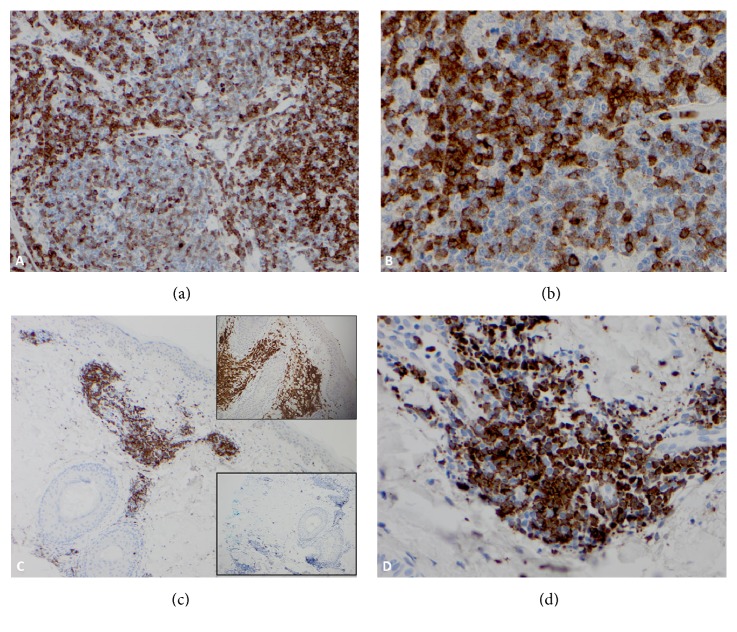
T-PLL involvement of lymph node (a, b) and skin (c, d). T-cells in the interfollicular areas are positive for TCL1 immunostain ((a), 200X and (b), 500X). T-cell aggregates in the dermis are positive for TCL1 ((c), 100X, (d), 400X). The insets in figure (c) show expression of CD3 (top) and lack of expression of CD20 (bottom) in neoplastic cells.

In light of the new diagnosis of T-PLL, the diagnostic tissues from original pelvic lymph node, obtained at the time of prostatectomy and from one of the skin biopsy specimens, were reanalyzed with TCL1 immunostain to evaluate if T-PLL was present at the time of diagnosis of CLL (Figures 5(a)–5(d)). Both the lymph node and the skin biopsy specimens showed the presence of T-PLL cells highlighted by strong nuclear and cytoplasmic TCL-1 expression.

**Figure 6:**
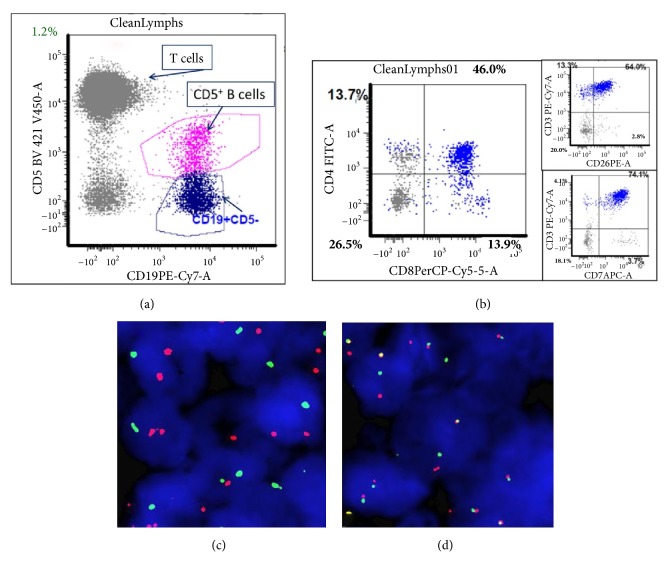
Composite CLL/SLL and T-PLL. Flow cytometry immunophenotype ((a) and (b)) of a bone marrow aspirate specimen. Panel (a) shows a prominent T-cell population (CD5+, CD19-) as well as a minor CD19+/CD5+ B-cell population that corresponds to CLL/SLL. Panel (b) shows that 46% of the analyzed bone marrow cells were CD4+/CD8+ corresponding to T-PLL, as detected in 25% of T-PLL cases. This lymphocyte population also expressed CD7 (bright and homogenous) and CD26, further supporting the diagnosis of T-PLL. FISH analysis ((c) and (d)): (c) FISH analysis with* ATM* (green) and* TP53* (red) exhibits 2 red, 1 green signal pattern, indicating* ATM* gene deletion; (d) FISH analysis with a* TCL1* break-apart probe exhibits 1 red, 1 green (split), and 1 yellow signal, indicating* TCL1* rearrangement.

Flow cytometry immunophenotype of the bone marrow showed that 46% of analyzed cells were positive for CD3, CD7 (bright), and CD26. Also identified were two monotypic B-cell populations with 1.2% of total cells being CD5-positive and 2.8% of total cells being CD5-negative (Figures 6(a) and 6(b)).

FISH analysis was also performed on formalin-fixed paraffin-embedded pelvic lymph node to evaluate for rearrangements of* TCL1* and* ATM. TCL1* rearrangement was mainly seen in the interfollicular area (Figures 6(c) and 6(d)), whereas* ATM* was detected in both follicular and interfollicular areas. Therefore,* TCL1* rearrangement was mainly confined to the T-cell component, while* ATM* deletion was detected in both the T-cell and B-cell components. Although desirable for a more definitive assessment of the mutations, cell sorting was not performed on analyzed specimens.

**Figure 7:**
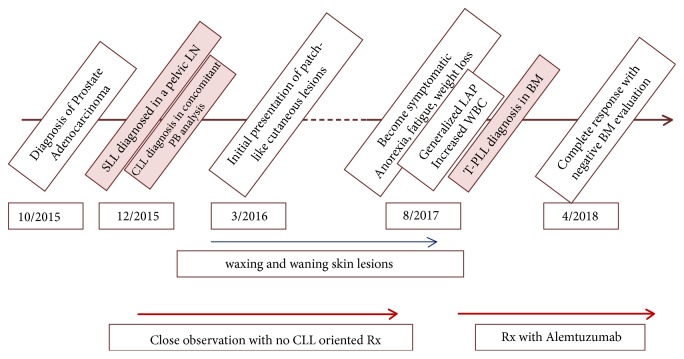
Timeline of most important pathologic findings from the diagnosis of prostate adenocarcinoma to T-PLL (abbreviations: BM: bone marrow, CLL: chronic lymphocytic leukemia, LAP: lymphadenopathy, LN: lymph node, PB: peripheral blood, Rx: treatment, SLL: small lymphocytic lymphoma, T-PLL: T-cell prolymphocytic leukemia, WBC: white blood cell count).

Figure 7 illustrates the chronological order of patient's diagnoses and clinical management.

## 3. Discussion

CLL/SLL is the most common type of leukemia in Western countries [12, 33]. Although usually indolent, about 3-8% of CLL/SLL patients undergo transformation to a higher-grade lymphoma or leukemia, commonly to a diffuse large B-cell lymphoma, known as Richter transformation. It is also well recognized that patients with CLL/SLL have an increased risk of developing secondary malignancies likely due to underlying immunodeficiency or therapy [34, 35]. The distinction between true Richter transformation and the presence of a second, different lymphoid neoplasm (composite lymphoma) is not always apparent [36, 37]. The detection of clonal relationship between CLL and the higher-grade lymphoma supports Richter transformation [38]. More rarely, patients with CLL are followed by T-cell lymphoma or leukemia; however, in most of these cases, a clonal relationship was not shown [27, 28, 30, 39, 40].

On the other hand, T-PLL at the time of initial diagnosis is usually an aggressive neoplasm with rapid rising, high-level lymphocytosis, organomegaly, constitutional symptoms, and frequent laboratory abnormalities [1, 3, 41]. However, there is a subset of patients with T-PLL who present with a more indolent clinical behavior and moderate, relatively stable lymphocytosis [42–45]. Patients with indolent disease usually have a WBC of about 20 × 10^9^/L (range: 10-50 × 10^9^/L) and they have better overall survival compared to the aggressive forms [4, 5, 46]. A subset of patients with apparently indolent T-PLL subsequently manifest with an aggressive phase of disease with poor prognosis.

Herein we report a rare example of composite CLL/SLL and T-PLL. Composite lymphoma/leukemia of B-cell and T-cell lineage has been rarely noted in the literature [25, 39]. T-cell neoplasms reported as part of composite lymphoma (or possible transformation) with CLL/SLL have been of cytotoxic CD8^+^ T cell immunophenotype and we are unaware of any reports of cases similar to the case we report. Incidental CLL/SLL found in lymph nodes evaluated for other reasons is not an uncommon event [47–50]; however, the incidental finding of T-PLL is rare [4]. The presence of CLL/SLL in the dissected lymph node, though in a rare follicular pattern [51], in conjunction with a CLL/SLL clone in the peripheral blood, led to inadvertent failure to diagnose the concurrent T-PLL in the same lymph node. After the diagnosis of T-PLL, the patient was treated with standard protocol anti-CD52 monoclonal antibody (Campath) with complete morphological response and negative flow cytometric and molecular measurable minimal residual disease (MRD) for both T-PLL and CLL/SLL. As alemtuzumab is an efficient therapy for both T-PLL and CLL, this result is not unexpected and may likely be used in rare cases of composite leukemias [8, 52, 53].

Ideally the neoplastic cells of CLL and T-PLL should be analyzed separately, for example, after flow cytometry based-cell sorting, for underlying genetic and molecular abnormalities to unequivocally detect the possible initiation from common progenitor cells for both CLL and T-PLL cells. Subsequent albeit retrospective FISH analysis on FFPE pelvic lymph node showed that* ATM* deletion involved both the CLL/SLL and T-PLL areas, whereas* TCL1* rearrangement was only detected in the interfollicular/T-PLL areas. These findings indicate that both CLL and T-PLL harbor* ATM *deletion and suggest that these two neoplasms could be derived from the same progenitor cells, a novel pathogenic mechanism in both neoplasms. This is in accord with a mechanistic role of* ATM* and* ATM/TCL1* abnormalities in pathogenesis of CLL/SLL and T-PLL, respectively [44, 54]. While abnormalities involving* ATM* gene are seen in approximately 20% of CLL/SLL patients, the prevalence is much higher in T-PLL observed in over 80% of patients [44, 55].

In summary, we report a rare case of composite CLL/SLL and T-PLL that highlights the importance of careful histopathologic and immunophenotypic features of hematologic neoplasms with overlapping morphologic and immunophenotypic features. We also highlight the use of FISH in tissue sections that may complement routine immunohistochemical studies in defining the distribution of diseases with specific cytogenetic abnormalities such as* ATM* deletion and* TCL1 *translocation. Lastly, the recently noted high frequency of* ATM* mutations in T-PLL is of interest, another feature that overlaps with CLL/SLL.

## Figures and Tables

**Table 1:** Summary of the most important clinical and pathologic features of chronic lymphocytic leukemia/small lymphocytic lymphoma (CLL/SLL) and T-cell prolymphocytic leukemia (T-PLL) as seen in our patient. Of note, these features do not necessarily represent the most common features of CLL/SLL or T-PLL.

Features	CLL/SLL	T-PLL
Initial presentation	Incidental enlargement of LN (pelvic LN dissection)	Generalized and constitutional symptoms

Immunophenotype	CD20+, CD5+, CD19+, CD23+, CD200+	CD3+, CD5+, CD7+, CD4+, CD8+, CD26+

TCL1 immunostain	Negative	Positive

*IGH/T*-cell gene rearrangements	Clonal *IGH*	Clonal *TRB, TRG*

Chromosomal and gene abnormalities	Trisomy 12 *ATM* deletion	inv(14)(q11.2q32) *ATM* deletion, *TCL1* rearrangement

NGS analysis	NM_000051.3(ATM):c.8078_8080del p.A2693del NM_002468.4(*MYD88*):c.794T>C p.L265P	
